# Tetany Due to Foreign Bodies in the Stomach

**Published:** 1905-01-28

**Authors:** 


### TETANY DUE TO FOREIGN BODIES IN THE STOMACH.

TETANY rarely occurs in adults apart from dilatation of the stomach, secondary to pyloric stenosis; and the condition follows immediately after vomiting or the passage of a stomach tube for purposes of investigation or lavage sufficiently often to suggest that irritation of the gastric mucosa plays an important part in producing the tetanic seizures, whatever other factors may also be necessary. This hypothesis is supported by a remarkable case reported by Dr. James Warbasse in the *Annals of Surgery*. His patient was a tailor who had been wont to entertain his fellows by swallowing nails, pins, and other small articles. As time went by and no evil results followed, it appears that he gradually took to swallowing larger bodies, and at last adopting the vocation of "a human ostrich," he became bolder still. All went well for three years when, after swallowing several nails and a watch chain, he suddenly became unconscious. He was soon well again, and continued to give exhibitions. Three months later, after swallowing two or three watch-chains, he had violent vomiting followed by unconsciousness and convulsions. He was taken to hospital, where he complained of epigastric cramps and had several convulsive seizures. A gastrotomy was successfully performed, and a large number of metal articles, including 129 ordinary pins, was removed. After his discharge from hospital he resumed his performances, without any apparent ill-effects, until two and a-half years afterwards, when he again began to have convulsive seizures. The attacks were repeated at intervals, and 10 months later a second gastrotomy was done, and 16 ounces of metal odds and ends, such as keys and knives, were extracted, and the patient recovered. Interesting points in the case are the absence of dilatation of the stomach, and the fact that all the attacks were preceded by vomiting, or a violent effort at vomiting.

